# An observational study protocol to explore loneliness and systemic inflammation in an older adult population with chronic venous leg ulcers

**DOI:** 10.1186/s12877-021-02060-w

**Published:** 2021-02-10

**Authors:** Teresa J. Kelechi, Robin C. Muise-Helmericks, Laurie A. Theeke, Steven W. Cole, Mohan Madisetti, Martina Mueller, Margaret A. Prentice

**Affiliations:** 1 College of Nursing, Medical University of South Carolina, Charleston, South Carolina USA; 2 Department of Regenerative Medicine and Cell Biology, Medical University of South Carolina, Charleston, South Carolina USA; 3 School of Nursing, West Virginia University, Morgantown, West Virgina USA; 4 Department of Medicine, University of California Los Angeles, Los Angeles, California USA; 5 Department of Public Health Sciences, Medical University of South Carolina, Charleston, South Carolina USA

**Keywords:** Loneliness, Chronic venous leg ulcers, Inflammation, Social genomics

## Abstract

**Background:**

Chronic venous leg ulcers (CVLUs) are the most common type of lower extremity wound. Even when treated with evidenced-based care, 30–50% of CVLUs fail to heal. A specific gap exists about the association between psychosocial stressors, particularly loneliness, and biomarkers of inflammation and immunity. Loneliness is highly prevalent in persons with CVLUs, has damaging effects on health, and contributes to the development of multiple chronic conditions, promotes aberrant inflammation, and diminishes healing. However, the confluence of loneliness, inflammation and the wound healing trajectory has not been elucidated; specifically whether loneliness substantially mediates systemic inflammation and alters healing over time. This study seeks to address whether there is a specific biomarker profile associated with loneliness, CVLUs, and wound healing that is different from non-lonely persons with CVLUs.

**Methods:**

An observational prospective study will identify, characterize and explore associations among psychosocial stressors, symptoms and biomarkers between 2 CVLU groups, with loneliness+ (*n* = 28) and without loneliness- (*n =* 28) during 4 weeks of wound treatment, measured at 3 time points. We will examine psychosocial stressors and symptoms using psychometrically-sound measures include PROMIS® and other questionnaires for loneliness, social isolation, depression, anxiety, stigma, sleep, fatigue, pain, quality of life, cognition, and function. Demographics data including health history, sex, age, wound type and size, wound age, and treatment will be recorded from the electronic health record. We will characterize a biomarker panel of inflammatory genes including chemotaxic and growth factors, vascular damage, and immune regulators that express in response to loneliness to loneliness and CVLUs using well-established RNA sequence and PCR methods for whole blood samples. In an exploratory aim we will explore whether age and sex/psychological stressors and symptoms indicate potential moderation/mediation of the effect of loneliness on the biomarker profile over the study period.

**Discussion:**

This study will provide insight into the influence of psychosocial stressors, symptoms, and biological mechanisms on wound healing, towards advancing a future healing prediction model and interventions to address these stressors and symptoms experienced by persons with CVLUs.

## Background

It is well established that individuals with chronic wounds, particularly those with chronic venous leg ulcers (CVLUs), have substantially reduced quality of life [1–3]. A wound is defined as chronic when it fails to reduce in size by 50% for at least 6 weeks after onset [4]. Up to 150 million individuals worldwide live with slow or non-healing ulcers. This is problematic because CVLUs account for ~ 80% of all chronic ulcers [5] that take months to years to heal [6]; for those that do heal, as many as 70% recur within 3 months [7].

Most CVLUs are associated with physical, psychological, and social symptoms. Physically, people with CVLUs experience symptoms such as odor, drainage, and leg swelling, which affect functional ability and physical appearance [8]. Self-management of CVLUs has been linked to^,^ fatigue and pain [9], and physical exhaustion [10] from the intense care required. Psychological symptoms reported by people with CVLUs include fear that the ulcer will never heal, will get infected, or that an amputation may occur [1, 10]. In the literature, cognitive dysfunction, poor self-image and worry [1], depression and anxiety have been reported [9]. These physical and psychological symptoms are known to negatively influence mobility, sleep, and mood, and limit professional, familial and social relationships, especially contact with friends [11]. Frequent and prolonged care is reported to lead to feelings of anger and resentment [12].

It is possible that sex, psychosocial stressors, and symptoms play a predominant role in perpetuating a chronic, non-healing state yet these factors are understudied, rarely assessed, and not consistently managed during clinical encounters. It is concerning that 30–50% of CVLUs fail to heal, even when treated based on best clinical practices which include: compression and leg elevation to improve venous blood flow, wound dressings to control necrotic tissue and wound exudate, and advanced topical biological therapies such as skin substitutes and growth factors to alter the local wound environment [13]. In the past 10 years, two studies have discovered that males with CVLUs report higher scores for both depression and anxiety [14, 15]. The findings regarding sex and associated symptoms underscore the critical need to explore the psychosocial and biological mechanisms that influence wound healing [16].

A specific gap exists when seeking knowledge about the association between psychosocial stressors, particularly loneliness, and biomarkers of inflammation and immunity. Loneliness has damaging effects on health, and contributes to the development of multiple chronic conditions [17], and diminished healing. Population-based longitudinal research indicates that loneliness predicts functional decline, morbidity and mortality, independent of objective social isolation, depression and health behavior [18, 19]. Loneliness is defined as a distressing feeling that accompanies the perception that one’s social needs are not being met by the quantity (social isolation) or especially the quality of one’s social relationships with individuals and/or the community [17, 20–23]. Loneliness affects ~ 50% of older adults in the U.S. [24], 68% of older adults with chronic wounds [25], and is associated with cardiovascular disease, hypertension, stroke, cancer, functional disability, and cognitive decline [26–28]. The negative health effects of loneliness exceed those of cigarette smoking, physical inactivity, and obesity [17, 29–31].

A growing body of evidence indicates wound healing is substantially altered by loneliness and social isolation in both human and animal models [32–34]. Loneliness is associated with altered molecular mechanisms such as inflammation and increased levels of pro-inflammatory cytokines [35], that may impede wound healing [36–38]. In addition, exposure to psychosocial adversity has been shown to elicit vascular, neuroendocrine physiological responses including increased peripheral resistance and dysregulation of the hypothalamic-pituitary adrenocortical (HPA) axis [38].

A growing literature in human social genomics suggests that psychosocial adversity and a negative social environment are associated with differential expression of hundreds of gene transcripts involved with inflammation and antiviral response, known as the *conserved transcriptional response to adversity* (CTRA) pathway. Loneliness has been associated with up-regulated inflammation by enhancing the transcription of genes such as interleukin-1 alpha and beta, interleukin-6, interleukin-8, cyclooxygenase 2, and tumor necrosis factor [39, 40] and the potent pro-inflammatory NF-kappa B gene [41]. The CTRA pathway is characterized by decreased expression of Type I interferon-related antiviral genes and increased expression of proinflammatory genes. However there is a paucity of literature to determine whether these same genes are also expressed in individuals with loneliness and chronic wounds [42]. The premise of this study is that a ~ 50-gene composite score previously used to assess the CTRA profile will be up-regulated in lonely (L+) individuals with CVLUs supporting the link between chronic wounds and inflammatory genes [43, 44]. The overexpression of these wound-related genes, in particular inflammatory cytokines may be considered candidates for the prediction of treatment response among patients with L+ and CVLUs. Experimental studies in animal and human models show a causal impact of adverse social conditions on CTRA gene expression [45]. Specific to chronic wounds, systemic inflammation is a well-established biological pathway linked to poor wound healing outcomes and is the focus of our study.

Recognizing that inflammatory biomarkers such as elevated cytokines and altered gene expression exist in CVLUs, we hypothesize that substantially heightened inflammation is a common molecular mechanism with a distinct profile that underlies both loneliness and poor wound healing in a chronic wound population compared to a wound population without loneliness. However the confluence of loneliness, inflammation and the wound healing trajectory has not been elucidated; specifically whether loneliness substantially mediates systemic inflammation and alters healing over time. The major question this study seeks to address towards advancing wound and symptoms sciences is whether there is a specific biomarker profile associated with loneliness, CVLUs, and wound healing that is different from non-lonely patients with CVLUs by addressing the following aims:

### Aim 1

Examine whether psychosocial stressors (i.e., social isolation, social support) and symptoms (i.e., fatigue, pain, depression, anxiety, sleep disturbance, reduced QOL) differ between (L+) and (L-) patients with CVLUs using well-validated questionnaires.

### Aim 2

Characterize a biomarker (chemotaxic factors, growth factors, vascular damage and immune regulators) profile common to loneliness and CLVU using well-established RNA sequencing and PCR methods for whole blood samples. We will start the analysis using a 1.8-fold change in gene expression, either positive or negative, to generate a composition score to assess the effect of loneliness and stressors on biomarkers.

### Exploratory aim 3

Explore whether age and sex/psychological stressors and symptoms indicate potential moderation/mediation of the effect of loneliness on the biomarker profile over the study period.

## Methods/design

### Overall strategy

The overarching aims of this observational prospective study are to identify, characterize and explore associations among psychosocial stressors, symptoms and biomarkers (panel of inflammatory genes that express in response to loneliness) between 2 CVLU groups, L+ (*n* = 28) and L- (*n =* 28) during wound treatment at 3 time points – baseline (V1), end of month 1 (V2) and end of month 2 (V3) - over a 2-month period. Eligibility criteria include: female and male; ≥ 60 years of age; CVLU of ≥6 weeks duration, and English speaking. Patients will be excluded if they are undergoing chemotherapy (affects immune factors), or taking steroids (may decrease inflammation and affect cytokine levels). We do not want to exclude participants based on wound treatment because the point of the proposal is to better understand loneliness in people with wounds. We are collecting treatment data but it will not determine inclusion. We also want to characterize participants by loneliness based on treatment but in order to do that, we will need to do a subsequent adequately powered study to determine the influence of treatments. Demographic and health data will be obtained from participants and the electronic health record. All participants will be recruited from the local wound communities/wound clinics in the southeastern region of the U.S.

**Fig. 1:**
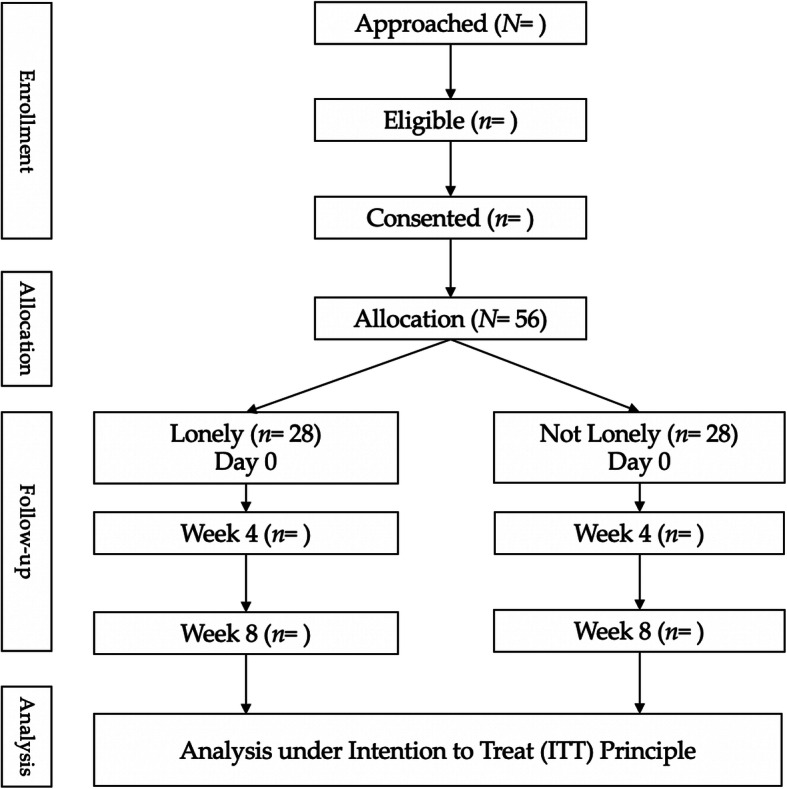
CONSORT diagram of participant flow in the study

Participant compensation will be provided. In return for their time and effort, each participant will receive a check for $25.00 for each completed study visit. Checks will be mailed to the address that the participant provides to the researchers. Additionally, we will offer a “fish bowl” prize drawing at each visit in which patients can “win” an additional $1 to up to $25 at V2 and V3 – we have used this contingency management strategy in our previous wound prevention studies with great success (7% attrition rate in a recently completed randomized controlled trial). The odds for the prize drawing are devised so that participants will not in all likelihood receive disproportionate amount of study compensation. A CONSORT diagram of participant flow through the study is provided below in Fig. 1.

### Demographics

Age, race, ethnicity, sex, marital status, education, disability status, income (socioeconomic status), and employment information will be collected by study personnel and recorded on the investigator-generated study specific demographics form developed for use in our previous studies. We will enter all data for the 3 visits into our study database in REDCap, a secure electronic documentation data storage management system. There are known sex differences in the neuroendocrine stress response and in the prevalence, symptoms, and correlates of loneliness in adults [46]. In our prior studies we reported that, in older adults, female sex was predictive of loneliness yet in rural areas such as Appalachia, mid-life men had higher mean loneliness scores [47]. Therefore, it is likely that loneliness impacts the health of males and females differently, making it critical to include sex as a biological variable in the study design. We will attempt to recruit equal numbers of males and females per loneliness group (L+ vs L-). Though no sex-specific hypotheses will be tested in the proposed study, we plan to explore possible differences of the effect of loneliness by sex (moderation) in the analyses, for example, through inclusion of sex-by-interaction terms). As this study is not powered to confirm hypotheses regarding moderating effects, these analyses will be considered hypothesis generating and descriptive rather than hypothesis testing. Per NIH guidelines, we will also report outcomes separately by sex.

### Instruments

The SPIRIT diagram (Table 1) below summarizes the schedule of enrollment, assessments, and visits across the study.

**Table 1:** Study SPIRIT diagram

TIMEPOINT	STUDY PERIOD		
	ENROLLMENT	VISIT SCHEDULE	
	Day 0	Week 4	Week 8
ENROLLMENT			
Eligibility screening checklist	X		
Informed consent	X		
Allocation (L+/L-)	X		
ASSESSMENTS AND MEASURES			
Demographics and characteristics	X		
Wound assessment and medical health history	X	X	
VEINES-QOL/Sym - 26 item			
PROMIS Cognitive Function 6a	X	X	X
PROMIS Global Health Scale - 10 item	X	X	X
PROMIS Anxiety 6a	X	X	X
PROMIS Fatigue 6a	X	X	X
PROMIS Pain Interference 6b	X	X	X
PROMIS Sleep Disturbance 6a	X	X	X
Katz Activities of Daily Living - 6 item	X	X	X
Mini-nutritional status - 7 item	X	X	X
UCLA Loneliness Scale - 20 item	X	X	X
Medical Outcomes Social Support Survey - 19 item	X	X	X
Patient Health Questionnaire - 9 item	X	X	X
The Stigma Scale: Body Image and the Skin - 8 item	X	X	X
Blood Sample: RNA for deep sequencing analysis*	X	X	X

*Chemotaxic factors: CCL2/JE/MCP-, CCL3/MIP-1 alpha, CCL4/MIP-1 beta, Granzyme B, CCL5/RANTES, CCL11/Eotaxin, CCL19/MIP-3 Beta, CCL20/MIP-3 alpha, CX3CL1/Fractalkine, PD-L1/B7-H1, CXCL1/GRO alpha/KC/CINC-1, CXCL2/GRO, and CXCL10

*Immune regulators: IL-3, IL-4, IL-5, IL-6, IL-7, IL-8/CXCL8, IL-10, IL-12 p70, IFN-alpha IL-13, IFN-beta IL-15, IFN-gamma IL-17/IL-17A, IL-1 alpha/IL-1F1, IL-17E/IL-25 CD40 Ligand/TNFSF5, IL-1 beta/IL-1F2, IL-33, IL-1ra/IL-1F3, and IL-2

*Vascular damage: VCAM, ICAM, CRP, MMP-1, MMP-3, and MMP-

*Growth factors: PDGF-AB/BB, TGF-alpha, EGF, TNF-alpha, FGF basic/FGF2 Flt-3 Ligand TRAIL, G-CSF, GM-CSF, VEGF, and PDGF-AA

#### Aim 1. Stressors and symptoms measures

In our previous trials, we tested the feasibility of the battery of instruments which took approximately 45 min to complete and found this was acceptable to our patients. However we are mindful of response burden and the time commitment for completing questionnaires and having blood specimens obtained. At the 3 data collection periods during regular wound clinic visits, participants can opt to answer questionnaires via assistance from study personnel in paper form or electronically on a tablet. Participants in both groups will receive the same battery of instruments. Psychometrically-sound measures include PROMIS® and other measures of loneliness, social isolation, depression, anxiety, stigma, cognition, and function. Data on wound type and size, wound age, and treatment will be recorded from the electronic health record. In the event that a participant expresses psychological distress when responding to psychological questionnaires (e.g., depression scale), our study wound physician will be contacted. We developed a plan for referral of depressed or suicidal participants.

#### Aim 2: blood draws for genomics analyses

Patient blood samples will be drawn by study personnel via venipuncture directly into PAXgene RNA tubes which inhibit RNA degradation and allow for purification of RNA for subsequent sequencing analyses. All blood samples will be drawn during the clinic visit, placed in the transport bag, immediately delivered directly to the MUSC laboratory at room temperature, and and slowly frozen to − 20 °C prior to processing, shipping, and analysis. RNA from patient samples will be sequenced in a blinded fashion in batches. We will attempt to schedule patients for clinic visits between 9 and 11 am to reduce the influence of diurnal variations.

### Feasibility and process monitoring

We will observe 10% of data collection procedures including questionnaires and specimen collection at least monthly. The team will track other study procedures through review of consenting logs, noting any problems encountered during interviewing and specimen analysis, participant burden of completing questionnaires/time taken, and document other barriers such as transportation that arise during data collection.

### Data analyses

Univariate descriptive statistics and frequency distributions will be calculated as appropriate for all variables. Inflammatory profiles, loneliness, psychosocial variables, and physical and functional variables will be obtained at baseline (V1), month 1 (V2) and month 2 (V3). Preliminary analyses will explore patterns of missing data for all outcome variables. We will also explore differences in demographic and other variables such as age (60–74, ≥75 years), sex, chronic illnesses, cognition, health status, functional activity, nutritional status, length of time to heal (healing trajectory), and wound treatment type across the 3 time points. All statistical analyses will be conducted using SAS Statistical Software Version 9.4 (Copyright© 2016 by SAS Institute Inc., Cary, NC, USA).

#### Analyses for aim 1

The primary variables for aim 1 are social isolation and social support (psychosocial stressors) and fatigue, pain, depression, anxiety, sleep disturbance, reduced QOL (symptoms). To examine differences in and variability of psychosocial stressors and symptoms over time between the L+ and L- groups, we will compare the longitudinal profile of the psychosocial stressors and symptoms (in individual models) between the groups over the 3-month study period using mixed effects models (MEM) analyses. These analyses will estimate the average change in the dependent variable within each group (L+ vs. L-) and individual change in variables for participants. MEM analyses allow for missing data, measurement of study subjects at different time points during the study, and time varying covariates. MEM can also take into account the effect of clustering, e.g., correlation of repeated measurements within one subject. The psychosocial stressor or symptom will be used as the dependent variable with group (L+ vs. L-), time, and time-by-group interaction as primary independent variables. The GLMM analysis approach allows for time-varying variables; we will also report separately whether and how many patients switch from L+ to L- or vice versa. Nutritional status, functional activity, and wound treatment will be investigated during secondary analyses through inclusion as covariables in the longitudinal models (individually). Further, frequency distributions of adverse events and serious adverse events will be obtained. Proportions within categories of adverse eventsand serious adverse events for the L+/L- groups will be compared via chi-square analyses.

#### Analyses for aim 2

All RNA sequencing will be performed at the UCLA Social Genomics Core Lab. RNA will be extracted from PAXgene tubes (Qiagen RNeasy), tested for suitable mass and integrity, and converted to cDNA libraries using a high-efficiency enzyme system (Lexogen QantSeq 3′ FWD). Normalized cDNA libraries will be sequenced using an Illumin HiSeq 4000 instrument, targeting > 10 million reads per sample. Reads will be aligned to the reference human transcriptome and quantified as gene transcripts per million total mapped reads using the STAR aligner. Transcript abundance data will be log2 transformed for analysis by linear statistical models quantifying differential gene expression as a function of key predictors (e.g., loneliness wound healing) while controlling for potential confounders, particularly age, sex, race/ethnicity, BMI, smoking history, and heavey alcohol consumption history. The CTRA will be measured by a pre-specified 53-gene composite score used in previous social genomics studies, [48, 49] including 19 gene transcripts involved in inflammation (i.e., *IL1B, IL6, IL8, TNF*), 34 gene transcript involved in innate antiviral responses (*IFNB, IFI-, MX-,* and *OAS-*family genes). Scores will be composed by z-score standardizing expression of each gene and summing the resulting values after sign-reversing the antiviral genes to reflect their inverse contribution to the CTRA profile.

#### Analyses for aim 3

The goal of aim 3 is to explore whether age and sex/psychological stressors and symptoms indicate potential moderation/mediation of the effect of loneliness on the biomarker profile over the study period. The composite score of the biomarker expression values will be used as the dependent variable for this analysis. Subsequently, to compensate for the limited sample size of this exploratory analysis, a bootstrapping procedure that resamples the data with replacement will be employed to provide more accurate estimates of variability (95% CIs). The analysis will involve a comparison of the composite score between L+ and L- using MEM as described above. Moderators will be explored through inclusion of an interaction term in the model, for example, age-by-loneliness. Potential mediators will be explored individually using MEM. Exploratory analyses will follow the modified procedure suggested by MacKinnon, Fairchild & Fritz [48] of the causal steps approach developed by Baron & Kenny [50].

### Wound healing trajectory definitions

A normal healing trajectory is considered a reduction in wound surface area of 10–15% per week, or at least 30% over a 2-week period. Wounds that fail to decrease in size by 30% over the first 2 weeks of treatment have a 68% probability of failing to heal within 24 weeks [51]. Ulcer area reduction at 2 weeks predicts failure to heal by 24 weeks in the venous leg ulcers of patients living alone. Overall time to closure depends on wound size and characteristics, patient age, nutritional status, comorbid conditions, adherence to treatment, and additional factors to be explored in this study. While there is no wound severity score for venous leg ulcers (unfortunately there is no staging or classification score for these types of wounds similar to staging criteria for pressure injuries), consensus on severity among wound care experts is emerging and is based on wound clinical characteristics as follows:

- Grade 3 = size > 10 cm^2^, infection/biofilm/osteomyelitis, ulcer > 4 weeks without change in size, high exudate, pain > 7 numeric rating scale (NRS), necrotic debris debris/fibrin covering > 50% of wound bed, odor, periwound inflammation/maceration, large lower extremity edema based on calf circumference - would require Level 1 treatment approaches (see below);
- Grade 2 = size 5–10 cm^2^, critically colonized, moderate exudate, pain 4–6 NRS, necrotic debris/fibrin 25–50%, moderate lower extremity edema - would require Level 2 treatment; and,
- Grade 1 = size < 5 cm^2^, light exudate, pain < 4 NRS, necrotic debris/fibrin < 25% or > 75% pink granulation tissue, mild lower extremity edema – would require Level 3 treatment.

Healing is determined by consistent reduction in size and symptoms and presence of epidermis covering 100% of wound bed. There are treatment differences depending on wound characteristics such as drainage, necrotic debris, leg edema, and infection. We will account for changes in grade and treatment by treatment types (levels) in our analyses as follows:

- Level 1: inelastic, multi-component, high compression [> 40 mmHg], mechanical/sharps debridement, antibiotics, advanced healing technologies such as skin substitutes, hyperbaric oxygenation;
- Level 2: moderate compression [30–40 mmHg], exudate management, enzymatic debridement; and,
- Level 3: moderate compression, moist healing environment.

#### Sample size considerations

With 28 participants per group we will have 80% power to detect an effect size of 0.6 standardized units effect size between the 2 groups, assuming 2 post-baseline time points; intra-class correlation no greater than 0.4; level of significance [α] = 0.05, two-tailed. Assuming standard deviation (sd) = 2, this translates into a raw effect size (difference in change scores) of 1.2. If the pooled sd is 5 units, the standardized effect size is equivalent to a raw detectable difference in change scores between the groups of 4.5 raw scale units. We will enroll 10 males and 10 females prior in each group so that we can complete thorough data analysis for outcome comparisons by sex. If necessary we will over enroll to achieve 28 participant per group at V3. The distribution of patients at one of our main wound centers is 60.0% male, 40.0% female; 60.0% African American, and 40.0% White with mean age 70 years.

### Data and safety monitoring

Considering the study design and specific procedures to be performed, the overall risk level for enrolled participants is considered to be minimal. Accordingly, this study will employ the use of an Independent Safety Monitor (Board cerified D.O.) who will be provided with real-time electronic access to participant data. This physician will be responsible for reviewing and grading adverse events, monitoring the study safety profile, and making recommendations to the Principal Investigor regarding study modification, termination, and continuance.

## Discussion

**Fig. 2:**
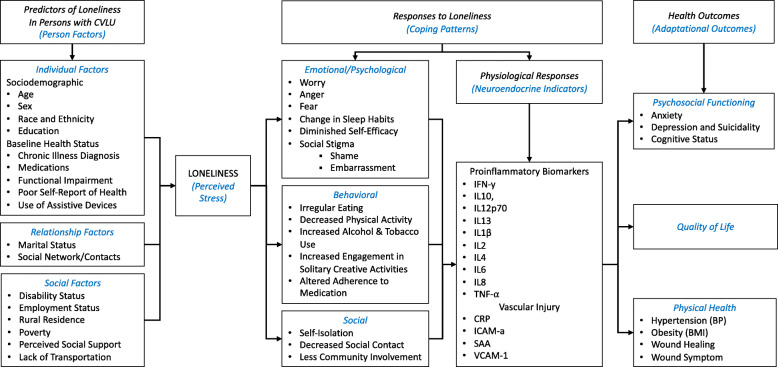
Conceptual model of Key Study Variables Mapped to Major Concepts of the Psychoneuroimmunological (PNI) Paradigm. Note: Variables included in this model were derived from results of preliminary studies and findings from the scientific literature on loneliness and chronic venous leg ulcers

There is a critical need to understand the trajectory of wound healing and interrelated psychosocial factors in lonely persons with CVLUs and to differentiate molecular mechanisms to advance future treatment approaches aimed to improve chronic wound healing and quality of life. We have demonstrated considerable expertise on the phenomenon of loneliness and its relation to health for chronically ill adults in the U.S. in analyses of Health and Retirement Study (HRS) data that identified predictors and outcomes of loneliness [52, 53] incorporated into the psychoneuroimmunological (PNI) conceptual model (Fig. 2).

Descriptive studies of loneliness in adults in Appalachia with chronic illness have reported loneliness correlated with depression (*r* = .388, *p* < .05), lower quality of life scores (*r* = .272, *p* < .05), and diminished social support (*r* = .274, *p* < .05), particularly diminished emotional support (*r* = .459, *p* < .01) [54]. Studies of stroke survivors discovered that loneliness was correlated *(p* < .05) with poorer quality of life on 13 domains from the Neuro-QoL [55, 56] and in systematic reviews, relationships were explicated between loneliness and multiple chronic conditions, providing evidence that loneliness may have higher prevalence in rural-residing individuals and that interventions are needed for loneliness in this population [17]. Further, recent qualitative work has discovered that the emotions of worry, anger, and fear are present for lonely older women and that lonely stroke survivors have unmet connection needs [47, 57]. A funded study led to the development of LISTEN, a novel intervention for loneliness that has been deemed feasible, acceptable, and initially effective for diminishing loneliness, and concurrently, diminishing measures of inflammation as persons became less lonely [58]. Finally, in a study of gene expression in lonely adults in a pilot randomized controlled trial of LISTEN, data suggest gene expression for inflammation and immunity changed in a favorable hypothesized direction [59]. The team spent extensive time identifying which variables to include, guided by the PNI model so that we could logically determine a gene expression profile in people with wounds, building on the current evidence base. Note also that previous research on gene expression in the HRS sample has also verified the association of loneliness with increased CTRA gene expression in older adults [60].

Previous research in social genomics has linked adverse social conditions to increased expression of pro-inflammatory genes in correlational human studies, in experimental animal and human models showing causal effects, and in cellular model systems dissecting the specific molecular signaling pathways involved [39, 43]. Loneliness in particular has been repeatedly linked to up-regulated expression of pro-inflammatory genes (i.e., *IL1B, IL6, IL8, TNF*), and down-regulated expression of genes involved in innate antiviral responses (*IFNB, IFIs, MX, OAS*) [18, 60–66]. This recurring pattern appears to reflect the activation of CTRA in myeloid-lineage immune cells [39, 67]. Our in-depth review of the literature guided the selection of these genes and is based on the best available data. We believe the novelty is in the application of the gene analysis to a population that suffers with wounds. However, the impact of this biomarker profile on chronic wound healing remains unstudied.

We have identified several challenges to our study including relevance of selected biomarkers, models to assess healing, sample size, generalizability of results, and racial/ethnic composition. Increased expression of inflammatory markers is associated with a variety of factors such as older age, multiple chronic conditions (obesity, diabetes), pain, smoking, lower socioeconomic status, and poor functional status [68–70]. We recognize the majority of individuals with CVLUs is generally older (≥60 years of age), has high functional impairment, poor quality of life, and multiple chronic conditions. However it is not known whether there is a distinct profile in patients with chronic wounds and in particular those with loneliness. We will account for these co-variates in our analyses.

Currently there is no assessment mechanism available to predict healing that includes an inflammatory biomarker or psychosocial variables such as loneliness and social isolation. If relationships are indicated among our study variables (and confirmed subsequently), these factors could be added to currently available disease-specific risk factor models to predict non-healing in wound populations. The M.A.I.D. score, predominantly used in wound care, was created out of 4 clinically-defined parameters to estimate long-term clinical outcomes [71]. However, there are no psychosocial, physical or biological components in this score, thus it lacks full scope risk factors for accurate prediction.

We recognize the small sample size may not allow for detecting statistically significant differences; however, as the focus of the exploratory aim is to generate hypotheses rather than to confirm, we will be able to establish preliminary relationship profiles among the psychosocial stressors and symptom variables. In addition, we will investigate whether there is an indication for clinically relevant differences that support further focused exploration in future studies.

We acknowledge generalizability of the anticipated results is limited in this small exploratory study and anticipate, in a future trial, we will have an adequately powered study and a greater diversity (Latinx) of participants. To this end, we will develop study materials, for example, in Spanish to reflect the diversity of the population.

The projected racial and ethnic composition of the participant sample is a concern given the established differences in gene expression among and between these groups; it is unlikely that the least frequent groups (e.g., Hispanic) can be properly evaluated in the proposed analyses, impacting on rigor. We agree and acknowledge the concern about the differences in gene expression among various ethnic/racial groups; for this small exploratory trial, we are focused on fidelity to our PNI model. Though it is not the primary aim of this observational study to determine ethnic differences, we have included age, sex, and race and ethnicity as individual person factors based soundly in the PNI model and these variables will be included and accounted for in preliminary analyses.

## Conclusion

Our study addresses a highly prevalent clinical problem - chronic venous leg ulcers (CVLUs) that affect millions of individuals worldwide, causing considerable suffering, disability and poor quality of life. The objective of this exploratory project is to assess stressors, symptoms and biomarkers associated with lonely and non-lonely individuals with CVLUs. Findings are expected to improve understanding of molecular mechanisms common to loneliness and inflammation towards development of a biopsychosocial prognostic indicator of healing potential in persons with chronic wounds. If differences in the biomarker profile of L+ individuals and CVLUs are found, further exploration of co-variates could form the bases of a new type of assessment that could potentially predict nonhealing.

## Data Availability

The datasets used and/or analyzed during the current study will be housed and stored at the Medical University of South Carolina in accordance with the academic institution’s Records Retention policy. Data will be made available from the corresponding author on reasonable request after publication of the results on the main research questions.

## Abbreviations

CVLUs: Chronic venous leg ulcers
CTRA: Conserved transcriptional response to adversity
HPA: Hypothalamic-pituitary adrenocortical
HRS: Health and Retirement Study
L-: Without loneliness
L +: With loneliness.
LISTEN: Loneliness Intervention using Story Theory to Enhance Nursing sensitive outcomes
M.A.I.D.: Palpable pedal pulses (I), Wound area (A), Ulcer duration (D), and Presence of multiple ulcerations (M)
MEM: Mixed effects models
NRS: Numeric rating scale
PNI: Psychoneuroimmunological
sd: Standard deviation
